# Cyberbullying Among Adolescents: Stakeholder-Driven Concept Mapping Approach

**DOI:** 10.2196/12683

**Published:** 2019-06-28

**Authors:** Megan Andreas Moreno, Nikita Midamba, Henry S Berman, Peter S Moreno, Mike Donlin, Erik Schlocker

**Affiliations:** 1 Department of Pediatrics University of Wisconsin Madison Madison, WI United States; 2 Seattle Children's Research Institute Seattle, WA United States; 3 Seattle Children's Hospital Seattle, WA United States; 4 Department of Continuing Studies University of Wisconsin-Madison Madison, WI United States; 5 The School Safety Center Office of the State Superintendent Olympia, WA United States

**Keywords:** cyberbullying, adolescent

## Abstract

**Background:**

Cyberbullying includes bullying behaviors on the Web; these behaviors are inconsistently measured and lack standardized definitions. The Uniform Definition of Bullying provides a consensus-based definition of bullying, and it highlights the need for an evidence-based definition of a model for cyberbullying.

**Objective:**

Toward understanding the key elements and constructs defining cyberbullying, the objective of this study was to develop a stakeholder-driven conceptual model of cyberbullying.

**Methods:**

Concept mapping is a validated research method that leverages both qualitative and quantitative approaches to integrate stakeholder input on complex topics. This process was used to develop a concept map and adapt it through participant input to a conceptual model. The validated concept mapping approach includes 5 steps: preparation, generation (brainstorming), structuring (sorting), representation (statistical analysis), and interpretation. We recruited stakeholder participants, including adolescents, as well as parents and professionals representing education, health, and the justice system. Analysis included hierarchical cluster analysis to develop a cluster map representing cyberbullying, followed by adaptation of that map to a conceptual model through qualitative participant feedback.

**Results:**

A total of 177 participants contributed to the concept mapping process, including 69% females, 50% adults, and 68% Caucasian, representing each of our stakeholder groups. A total of 228 brainstorming items were generated and sorted into a concept map that included 9 clusters. Clusters included topics that had strong overlap with traditional bullying, such as consequences for perpetrators and targets, with example items “alienating” and “crippling.” Some clusters were unique, such as cyberbullying techniques, with example item “excessive messaging,” and characteristics of the cyberbullying experience, with example item “constant.” Through the interpretation step, a conceptual model emerged, illustrating connections and distinctions between traditional bullying and cyberbullying.

**Conclusions:**

We found that in generating a stakeholder-driven concept map of cyberbullying, participants could not describe cyberbullying without integrating key concepts from traditional bullying. On the basis of our conceptual model, there are unique characteristics of cyberbullying that suggest that uniform definitions of bullying need to be evaluated to ensure their application to cyberbullying.

## Introduction

### Background

Bullying is both a public health and a criminal justice problem that occurs throughout the world, and bullying can happen at many stages in the life course, from childhood to adolescence, even into adulthood. Although traditional “schoolyard” bullying remains problematic, over the past decade, technologies have provided new platforms on which bullying can occur. It is estimated that 19.6% of children of ages 14 to 18 years were bullied on school property, and 14.8% of children aged 14 to 18 years were electronically bullied [1]. These electronic forms of contact may include social networking websites (eg, Instagram, Twitter), Web-based games, instant messaging, short message service text messaging, and mobile phone pictures. This phenomenon has come to be known as cyberbullying. It is estimated that 1% to 41% of US adolescents have perpetrated cyberbullying and 3% to 72% have been targets [2].

### Consequences of Bullying and Cyberbullying

Previous studies have examined the substantial negative effects that cyberbullying can have on both targets and perpetrators. Adolescents who have experienced cyberbullying report higher levels of depression and lower self-esteem [3]. Furthermore, emotional distress, anger, sadness, detachment, externalized hostility, and delinquency are more common in targets of cyberbullying than in the general population [2]. Many of these effects are also seen in targets of traditional bullying, suggesting similarities in the negative consequences of these phenomena [4].

### Current Challenges in Understanding Cyberbullying

Assessing the prevalence of cyberbullying remains challenging, partly as the field lacks a conceptual approach or an operational definition of the term [5]. A consistent definition can support tracking of cyberbullying over time, and it has been called out as one of the major challenges in the field [6]. In the realm of traditional bullying, collaboration across experts in the field led to the development of a consensus-driven definition. Led by the Centers for Disease Control and Prevention (CDC), the Uniform Definition of Bullying is as follows: Bullying is any unwanted aggressive behavior(s) by another youth or group of youths who are not siblings or current dating partners; bullying involves an observed or perceived power imbalance, and it is repeated multiple times or is highly likely to be repeated. Bullying may inflict harm or distress on the targeted youth, including physical, psychological, social, or educational harm [7]. Within this definition, 4 different types of bullying behavior are commonly identified: physical, verbal, relational, and damage to property [6]. Observational studies have shown that the different forms of bullying of youths may overlap [8,9]. Within the CDC definition, cyberbullying is considered bullying by digital electronic means. Thus, cyberbullying is considered a context in which bullying occurs.

The extent to which the Uniform Definition can be applied to cyberbullying remains uncertain. A previous study that used focus groups with college students to discuss whether the Uniform Definition applied to cyberbullying found that students were wary of applying the definition. Participants in this study described elements of cyberbullying that they felt were distinct from the Uniform Definition, including their perception that cyberbullying often involves less emphasis on aggression, intention, and repetition than other forms of bullying [10]. A conceptual model describing key elements of cyberbullying could contribute to understanding the key components of cyberbullying and assessing how it may be similar or different compared with traditional bullying. A data-driven conceptual model could potentially provide evidence to inform definitions, measurement approaches, or future interventions.

### Study Purpose

Thus, the purpose of this study was to develop a stakeholder-informed conceptual model for cyberbullying. To fulfill this purpose and ensure the model was driven by participant views and data, concept mapping methodology was applied. This validated methodology has been applied toward developing conceptual frameworks to describe complex topics [9-11]. Previous concept mapping studies have been applied to complex topics, such as intimate partner violence, physical rehabilitation experiences, and adolescent sexuality [12-14]. This method has also been used in previous health research to provide insights into mental health and illness [10,12,14,15]. The outcome of this process is a concept map, a visual representation of the key concepts and their interrelationships. The final map that is created is entirely in the language of the participants, and it produces an easily interpreted visual representation that can be adapted to represent a conceptual model.

## Methods

### Study Setting and Design

This study was conducted in Washington State, and this study recruited participants from academic and community settings. The study design was concept mapping. The Western Institutional Review Board approved this study.

### Participants and Recruitment

The concept mapping approach is ideally suited for data collection from stakeholders relevant to the concept under investigation. To ground our conceptual framework in views of stakeholders involved in cyberbullying, participants included adolescents and young adults, aged 12 to 21 years, as these youth are those who directly experience cyberbullying. We also included parents of the youth of these ages, as parents are often involved when their children are cyberbullying. In selecting additional stakeholders for this study, we considered the evidence that cyberbullying can occur at home, at school, and in the community [16,17]. Thus, we included educators, including teachers and administrators. We also included professionals typically involved in cyberbullying prevention and identification or intervention: health professionals, such as physicians, nurses, social workers, researchers and counselors, and professionals involved in law and policy, including attorneys. Additional eligibility criteria included English speaking. Concept mapping studies typically use qualitative-sized samples of approximately 50 to 80 participants in total [9], often with higher numbers of participants at the data generation and interpretation steps (brainstorming and interpretation steps). As we had several stakeholder groups involved in this study, we planned to include a larger number of participants to ensure we achieved stakeholder representation across each concept mapping step and across the number of groups involved in this study [11]. All participants were recruited through purposeful sampling from academic and community organizations between March 2013 and December 2015. Purposeful sampling included contacting local schools, parent organizations, and universities to identity participants. Each adult participant gave written consent for participation; parental consent and adolescent assent were obtained for youth participants. Before the start of each data collection, participants completed a survey that included questions about age, gender, race/ethnicity, and role (ie, student, professional, and parent). Participants who completed the survey were provided a US $5 incentive, and participants who completed all other stages of data collection were provided a US $20 incentive.

### Concept Mapping

The concept mapping methodology was chosen, as it directly involves participants and balances group consensus with individual contribution, as some steps require group participation, whereas others are done individually. The method also allows for the consolidation of key concepts from a broad array of initial data points. A total of 5 steps are involved with the concept map creation process: preparation, generation, structuring, representation, and interpretation [9]. As we wanted to use our concept map to and wanted it to translate to a draft conceptual model, we also added a final step to propose and get feedback on a conceptual model.

#### Preparation

The goal of preparation was to develop a focus prompt to encourage brainstorming statements from participants in the generation step. The prompt was specifically designed to be an open-ended question that required participants to complete a sentence to achieve consistent phrasing. We developed a focus prompt of “A behavior or characteristic of cyberbullying is...”. This prompt was pilot tested with a convenience sample of adolescents, researchers, and health care providers before its use for data collection.

#### Generation (Brainstorming) Sessions

The goal of the brainstorming step was to generate a list of participant-generated items with sufficient breadth and depth to represent the full spectrum of ideas related to what defines cyberbullying. The concept mapping literature describes 2 approaches to collect brainstorming responses: Web-based survey and focus groups. To develop a brainstorming list with sufficient breadth and depth to inform our concept map, we used both approaches. First, individual brainstorming responses were conducted using a secure Web-based survey tool. The goal of the Web-based brainstorming approach was to allow for greater reach in participant sampling among the adult professional population. Second, brainstorming was conducted using a semistructured focus group format. Focus groups allowed for interaction among participants, as well as opportunities for participants to build on each other’s thoughts [18]. Each focus group included 5 to 8 participants and lasted between 45 and 90 min. Focus groups with youths were held separately from focus groups with adults. During focus groups, after obtaining consent and providing instructions, the facilitator presented the focus prompt to the group. Participants were initially given 10 min to write individual responses to the prompt on paper. Thereafter, the topic was opened for group discussion toward further idea generation and revision. At the conclusion of the session, all written responses were collected from the participants; any additional ideas that were discussed by the group as a whole were recorded by the facilitator through transcription of the audio recording. All focus groups were audio recorded and transcribed verbatim. A total of 2 investigators reviewed the transcripts to identify items to contribute to the brainstorming list. The brainstorming list was reviewed by 2 investigators to eliminate redundancy, and it was compiled into 1 revised list, representing all ideas and statements generated by the brainstorming step.

#### Structuring (Sorting) Sessions

The goal of the structuring step was to sort the statements generated in the brainstorming step. To form overarching constructs, this process provides insights into how individual ideas are related. In the sorting step, participants were given a stack of index cards, each of which had a single written item from the revised brainstorming list. Individuals were asked to sort the cards into categories that made sense to them and create a label for each pile. All groups were determined by the participants, each item could be sorted into only 1 group, and every group needed at least 1 item within it.

#### Representation

The goal of representation was to apply quantitative approaches to analyze the data toward creation of a visual point map representing individual items. Analyses were conducted using the Concept Systems Core software Build 2016.062.11 (Concept Systems Inc) and SAS software version 9.3 (SAS Institute). Sort data were organized into a square symmetric-similarity matrix (SSSM) for each participant. In this process, pairs of brainstorming ideas were tested to determine if they had been grouped together. An overall SSSM was constructed by summing the matrices for all participants. Multidimensional scaling (MDS) of the overall SSSM was used to produce a 2-dimensional point map [19]. The point map represented all items in a 2-dimensional plane; items that were commonly grouped together were closer together on the point map. Items that were rarely or never grouped together were further apart on the point map. Stress index was calculated to assess the fit of the MDS solution to the data. Stress indices ranging from 0.10 to 0.35 indicate acceptable fit for concept mapping, with lower values indicating better fit [9,12]. The cluster map was created by applying hierarchical cluster analysis over the overall SSSM. During this step, the software analyzes the data to perform cluster analysis and MDS to create a visual representation of the ideas in the form of clusters. The analysis process groups the ideas according to the results of the MDS into clusters. Items that were similarly categorized by participants appear closer together on the map than items that were not commonly categorized together. We then reviewed the point map overlaid with a range of clusters generated by the Concept Systems software. The role of the investigators was to use a consensus-driven iterative process to identify the cluster arrangement with the strongest theoretical support. Using the point map, the concept mapping software generates sequential versions of the concept map with a change of 1 cluster per version. The upper bound of the range of cluster arrangements examined was 2 SDs above the mean number of statement groups produced during the sort process. The lower bound of the range of number of clusters was the minimum number of clusters created by any participant. The analysis process included reviewing cluster arrangements sequentially and identifying the optimal cluster solution through an iterative process. Finally, the investigators assigned a label to each cluster on the basis of the theoretical construct described by its constituent statements. Each cluster was initially named by the software on the basis of the ideas generated by participants; names were reviewed and revised for clarity by 3 raters. The draft concept map was reviewed by all investigators to ensure it was qualitatively consistent and logical. Any revisions to the map were based on consensus of the investigators.

#### Interpretation

The first goal of these sessions was to conduct focus groups to allow participants to view, discuss, and interpret the concept map. The discussion was led by a facilitator and began with an introduction and review of the concept mapping methodology. The steps of the project and the focus prompt were reviewed; thereafter, the preliminary concept map was introduced. Participants were asked to discuss cluster groupings and labels, as well as to explore the overall structure of the map. Each group was asked ways in which the map represented the definition of cyberbullying and the ways it could be improved. After concluding focus groups, the data were transcribed verbatim and evaluated by 2 investigators. All focus groups were analyzed for comments reinforcing elements of the concept map, as well as suggesting edits to the map. A total of 2 investigators identified areas of consensus and used these to modify the concept map. If consensus was not reached via 2 investigators, a third was asked to review data and determine a decision on whether to modify the concept map. Following finalization of the concept map, the investigators then identified feedback specific to a conceptual model. The draft conceptual model was developed on the basis of feedback from participants in the interpretation groups. Similar to the abovementioned, 2 investigators identified areas of consensus and used these to modify the model. If consensus was not reached via 2 investigators, a third was asked to review data and determine a decision.

#### Translation to a Conceptual Model

We concluded focus groups in the interpretation step by asking about the transition to a conceptual model. We wanted to ensure the conceptual model was representative of the concept map and get input on the transition to such a model. Using a draft conceptual model, we then conducted a final series of key informant interviews to obtain feedback on both the concept map and the conceptual model to ensure alignment. These interviews were also recorded and transcribed verbatim. A total of 2 investigators reviewed transcripts. They identified areas of consensus around elements of the conceptual model and proposed edits to the model to reflect participant feedback.

## Results

### Participants

A total of 177 participants contributed to the study. A total of 80 participants contributed the generation step; this included 37 Web-based survey participants and 43 focus group participants across 6 groups. In the structuring session, 26 participants completed sort activities. In the interpretation step, a total of 71 participants contributed to a focus group or key informant interview. Youth had an average age of 17 (SD 2.25), and adults had an average age of 43 (SD 12.9). There were 50% of adults over age 21; adult professionals included 24% health professionals, 22% clinical researchers, 12% educators, and 2% attorneys. Table 1 provides demographic information of our participants across the concept mapping process.

**Table 1:** Participant demographics across steps of the concept mapping process.

Demographics of participants across concept mapping steps		Step 2: Brainstorming *focus groups* (n=43)	Step 2: Brainstorming *surveys* (n=37)	Step 3: Sorting *activity* (n=26)	Step 5: Interpretation *focus groups* (n=63)	Translation to conceptual model *key informant interviews* (n=8)	Total
**Gender, n (%)**							
	Female	34 (79)	32 (86)	17 (65)	34 (54)	5 (62.5)	122 (69)
	Male	8 (19)	5 (14)	8 (31)	29 (46)	3 (37.5)	53 (30)
	Unknown	1 (2)	0 (0)	1 (4)	0 (0)	0 (0)	2 (1)
**Age, n (%)**							
	Adults over 21 years of age	5 (12)	35 (95)	19 (73)	21 (33)	8 (100)	88 (50)
	Youth aged 21 and under	21 (49)	0 (0)	6 (23)	37 (59)	0 (0)	64 (36)
	Age unknown	17 (40)	2 (5)	1 (4)	5 (8)	0 (0)	25 (14)
**Race/ethnicity, n (%)**							
	Black/African American	3 (7)	2 (5)	2 (8)	4 (6)	1 (12)	12 (7)
	Asian/Pacific Islander	1 (2)	8 (22)	2 (8)	11 (17)	0 (0)	22 (12)
	Caucasian	31 (72)	25 (68)	20 (77)	40 (64)	5 (64)	121 (68)
	Hispanic/Latino	2 (5)	1 (3)	1 (4)	2 (3)	1 (12)	7 (4)
	Native American	3 (7)	0 (0)	0 (0)	4 (6)	0 (0)	7 (4)
	Mixed race	1 (2)	0 (0)	0 (0)	3 (4)	1 (12)	4 (2)
	Other/unknown	2 (5)	1 (3)	1 (4)	0 (0)	0 (0)	4 (2)
**Role, n (%)**							
	Student	22 (51)	1 (3)	5 (19)	28 (44)	0 (0)	56 (32)
	Health professional	13 (30)	8 (22)	1 (4)	15 (24)	2 (25)	39 (22)
	Educator/teacher	3 (7)	1 (3)	5 (19)	0 (0)	0 (0)	9 (5)
	Administrator/librarian	0 (0)	9 (24)	0 (0)	0 (0)	0 (0)	9 (5)
	Researcher	0 (0)	13 (35)	6 (23)	14 (27)	6 (75)	40 (24)
	Social worker	0 (0)	2 (5)	0 (0)	1 (2)	0 (0)	3 (2)
	Counselor	0 (0)	3 (8)	1 (4)	1 (2)	0 (0)	5 (3)
	Law professional	0 (0)	(0)	3 (12)	0 (0)	0 (0)	3 (2)
	Other/unknown	5 (12)	0 (0)	5 (19)	3 (3)	0 (0)	13 (7)
**Parent/nonparent, n (%)**							
	Parent	4 (9)	0 (0)	13 (50)	7 (12)	2 (25)	26 (15)
	Nonparent	0 (0)	0 (0)	11 (42)	52 (83)	6 (75)	63 (35)
	Unknown	39 (91)	37 (100)	2 (8)	10 (0)	0 (0)	88 (50)

### Step 2: Generation

A total of 311 statements were produced during the generation step of data collection. Refining the statement list led to removing of duplicate statements (n=18) and merging of similar statements (n=65). The final list of brainstorming statements included 228 unique aspects of cyberbullying.

### Step 3: Structuring

During the sorting procedure, participants sorted the statements into individual groups, the number of groups ranged between 4 and 30 individual groups (mean 12.9, SD 6.1, median 11).

### Step 4: Representation

**Figure 1:**
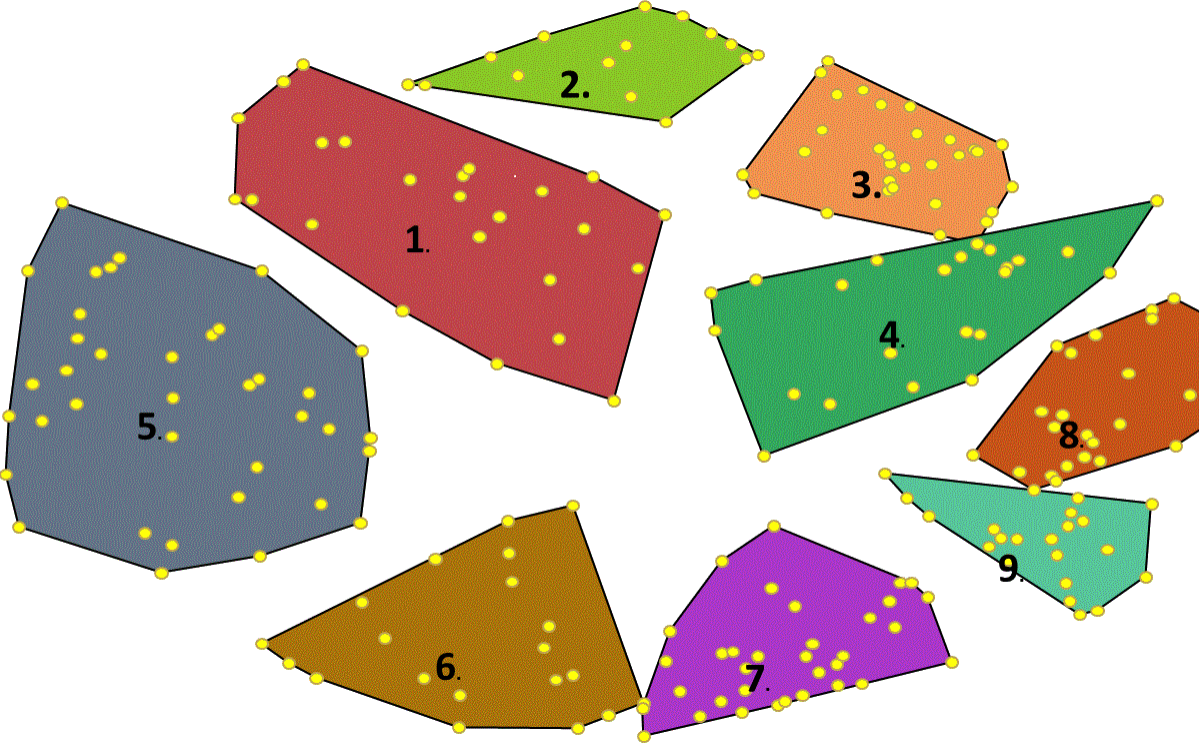
Cyberbullying concept map developed by stakeholders including adolescents and young adults, parents, community members such as educators, clinicians and attorneys. Each number represents a single item proposed by participants, and the clusters represents how participants sorted items into groups of alike concepts.

The stress value for the fit of the MDS solution to the structuring data was 0.3 for the 9 cluster solution, indicating adequate fit. Overall, the 9 cluster solution presented in Figure 1 was found to represent the best fit for the data after assessing a total of 10 unique cluster solutions, ranging between 2 and 12 clusters. The 9 clusters depicted on the Cyberbullying Concept Map are described in Table 2.

**Table 2:** Cyberbullying concept map clusters.

Cluster number	Proposed name	Example items
1	Characteristics of perpetrators and targets	Lack of empathy, afraid to go back to school, and “small minds”
2	Consequences for perpetrators and targets	Alienating, crippling, and devastating
3	Characteristics of the bullying experience	Aggressive, intent to harm, disrespect, and hostile
4 and 8	Bullying techniques	Ostracize, antagonize, and “mean girls”
5	Characteristics of the cyberbullying experience	Anonymous, constant, and perceived lack of consequences
6	Cyberbullying techniques	Making unwanted posts go viral, excessive messaging
7	Cyberbullying cases	Sending rude messages from someone else’s account to get people mad at the person
9	Perceived vulnerabilities	Negative statements about clothes, family situation, intelligence, social status, appearance, and sexuality

### Step 5: Interpretation

The interpretation step first involved reviewing the concept map and discussing participant perceptions of that map. Discussions by participants consistently centered on how to describe cyberbullying as a phenomenon that was perceived as both similar to and distinct from traditional bullying. Common topics of discussion included that many characteristics and motivations for bullying were considered to be similar and sometimes identical for both cyberbullying and traditional bullying. However, participants felt strongly that there were unique aspects to cyberbullying, including novel methods or situations in which bullying could arise, as well as providing new tools for bullying perpetrators. For example, participants described that a unique aspect of cyberbullying was that cyberbullying situations could arise from innocuous comments on the Web that are taken out of context or jokes that go too far. These messages can be virally spread, such that they then represent bullying. As an example, an adolescent described how compliments posted on the Web can be twisted to become “backlash compliments, like oh your hair looks *great* [emphasis added].” The adolescent further described that these sarcastic comments were more likely to be “liked” or “shared,” allowing them to be seen and disseminated by others. Another example described by a parent was learning that her son’s school was having a Web-based “draft,” described as follows: “they are actually, like, doing a draft, a first round draft, about which girls they want to take to the prom and ranking them, it is all done online.” It was described that this scenario may not be considered a traditional bullying situation but that it could have similar negative impacts on youth who were the targets. In these scenarios, the initial communication or situation may not have been unwanted or aggressively hurtful, but the situation could devolve into bullying because of the format of Web-based communication. Another area in which participants noted unique aspects of cyberbullying was how the Web-based environment provides tools so that a target of bullying can “turn the tables” to become a perpetrator. A quote from an adolescent described the following:

...cause when you’re in person you can see the physical build of the person and if they’re bigger than you, you don’t usually want to pick a fight with them. But on the internet, it’s just a screen in front of you with a username and they’re all the same that way.

This quote describes participants’ views of how a target of bullying can achieve power by using a “screen in front of you” to bully his/her perpetrator. Participants frequently discussed their perceptions of heightened fluidity of the roles of perpetrator and target in cyberbullying situations. The interconnectedness of traditional in-person bullying and cyberbullying was also a common topic. One example quote from an adolescent described the following:

Umm so the people I know that, or the people that I’ve known that have been cyberbullied usually they’re the targets from like bullying at school and they go and try to pass the pain on the internet to someone else, so it’s kind of like a circle going around like that cause they can’t like, they’re not like, the smaller guy can’t beat up the bigger guy, so he goes on the internet and destroys him on the internet, and the bigger guy comes back and destroys the little guy at school, so it’s just like a circle between the two.

A notable trend in participant contributions to the topic is that so many spoke from personal experiences with cyberbullying scenarios. Adolescents spoke about situations they or their peers had experienced. Parents often shared situations their children had experienced. A parent described the following:

I wrote down some based on, um, experience one of our daughters had about a year ago. Uh, repeated contacts that were unwanted, so, just continuous contacting, right, um, when not asked for, attempt to push beliefs on to the recipient that are not those of the recipient, uh, profane language if, of course it’s not wanted, uh, yelling in the electronic message if that’s not the, ya know, normal tone of the message, and just threats or blackmail.

### Translation to a Conceptual Model

**Figure 2:**
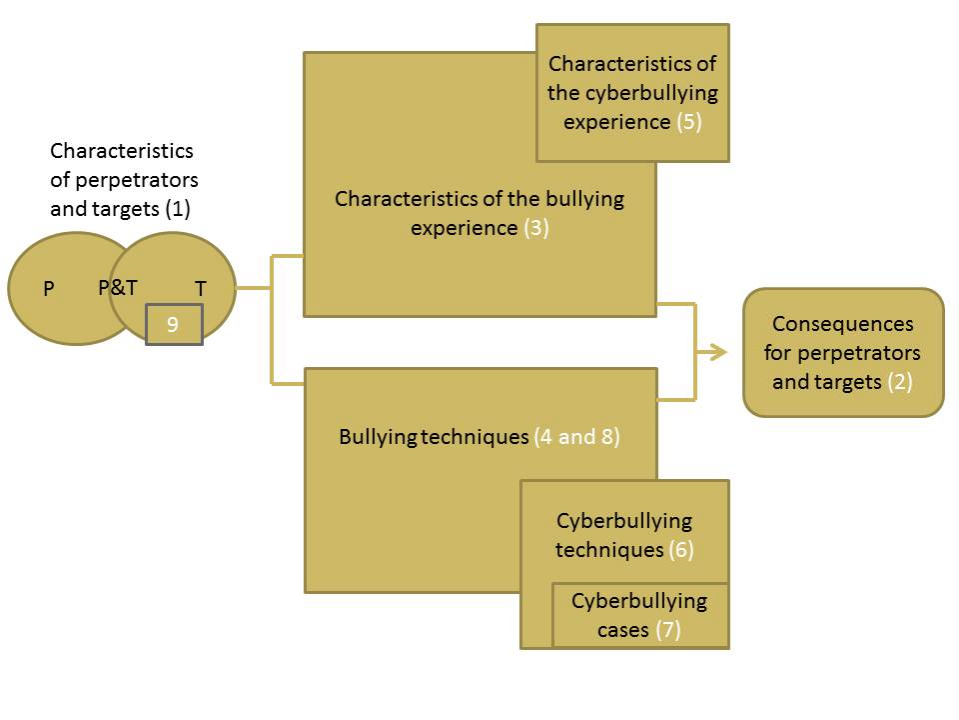
Conceptual model of cyberbullying and its relationship with bullying. Cluster numbers on the diagram are from cyberbullying concept map in parentheses.

On the basis of participant input, the conceptual model of cyberbullying included the relationship between cyberbullying and bullying (Figure 2). Key aspects of the conceptual model included the overlap in bullying perpetrators and targets, which includes clusters 1 and 9 from the concept map. Important characteristics of bullying perpetrators suggested by participants included bullying as “a way to deal with insecurities,” which was suggested by a teacher. A characteristic of bullying targets nominated by a legal professional was “afraid to go back to school.” Within the circle describing bullying targets were specific characteristics that were nominated as denoting particular risk for bullying, including being of racial or sexual minority groups. Some shared characteristics of both perpetrators and targets included “depression risk,” suggested by several participants, including adults and adolescents. The conceptual model also included 2 overlapping boxes, with the larger describing characteristics of the bullying experience; these included descriptors, such as disrespectful, mean, and aggressive. Overlapping this box was a smaller box representing unique characteristics of cyberbullying proposed by participants, such as “hides behind screen” (adult, parent). Similarly, a larger box described bullying techniques, including false information, public shaming, or belittling. Overlapping this box was a smaller box representing techniques that were specific to cyberbullying, including “displaying negative images” (adult, health professional) “covering with false names” (adult, social worker), and “virtual clique” (adult, administrator). Nestled within this box was a smaller box in which specific examples of cyberbullying cases were described, including “photo-sharing without consent” (adult, parent). The construct describing consequences to bullying perpetrator and targets was a shared construct. Participants reflected that they did not perceive specific differences between cyberbullying and traditional bullying for this construct. An area of discussion in which there was a lack of consensus was whether the concept map or conceptual model appropriately represented the role of bystanders. A quote from an older adolescent was, “I feel like this entire thing is just focused on the bully and the victim and not just, it’s just on them, and not the bystanders.” However, other youth discussed viewing cluster 4 on the concept map as adequately representing the role of bystanders.

## Discussion

### Summary

This study used a concept mapping approach to gain insights and perspectives from stakeholders toward a concept map and a stakeholder-driven conceptual model of cyberbullying. During the brainstorming step, stakeholders generated a diverse and expansive list of statements describing cyberbullying. The sorting procedure yielded a robust concept map of 9 clusters that comprised characteristics of the people involved, actions, and consequences that define bullying and cyberbullying. After our concept mapping process, we utilized stakeholder insights to develop a conceptual model that illustrates areas in which cyberbullying is similar to and unique from traditional bullying. This conceptual model represents participants’ perception of cyberbullying and suggests that cyberbullying can be best understood within the context of all bullying behavior, with recognition of the unique challenges it presents.

### Principal Findings

A major finding of this study is the several ways in which bullying and cyberbullying were aligned. This finding is aligned with emerging literature supporting strong connections between bullying and cyberbullying. Recently studies have illustrated that individuals involved in bullying often experience different types of bullying within a given situation, which may include verbal, physical, and cyber experiences [18,20]. Similarly, we found that a key area of overlap between cyberbullying and traditional bullying includes characteristics of the individuals involved. This study’s participants described characteristics of both bullying perpetrators and targets that applied to traditional bullying and cyberbullying, including describing bullying as a way to address insecurities. Participants emphasized the fluidity of roles between perpetrator and targets for both cyberbullying and bullying. They noted that an adolescent’s ability to engage in cyberbullying would not be limited by physical or social power; thus, cyberbullying may augment the fluidity of roles between perpetrator and target. This fluidity in roles is supported by Olweus’ descriptions of “the bullying circle” in which targets may become perpetrators (and vice versa) depending on situations and circumstances [21]. The Uniform Definition of bullying describes that bullying behavior involves an actual or perceived power imbalance. In this study, the fluidity in roles of perpetrator and target does not seem to represent a shift in the actual power of the individual, but it could represent power derived from the tool that is used to bully: the internet. Another area of similarity between cyberbullying and traditional bullying was that the consequences of both were described in a single construct in the interpretation diagram. This single construct implies that our diverse stakeholders, including educators, legal experts, health professionals, as well as teens themselves, perceive that significant and similar negative consequences result from both cyberbullying and traditional bullying. A second critical finding is the areas in which stakeholders elucidated their perceptions of differences between cyberbullying and traditional bullying. These included characteristics of the bullying experience, including the capacity for anonymity by “hiding behind screens” in cyberbullying. The role of anonymity in cyberbullying has been noted in previous studies [2]. However, traditional bullying is not without the capacity for anonymous actions, including sending threatening notes anonymously or damaging property secretly. Even so, the perceptions of participants about anonymous bullying via the internet was a topic of concern and even alarm for many participants. Finally, the concept map and accompanying conceptual model serve as data-driven visual representations of the complexity of bullying. This complexity is illustrated in our concept map, and it includes shared characteristics among perpetrators and targets, a variety of tools and approaches to consider, and negative consequences for both actors. Our findings support a need for research that considers mechanisms or processes that can explain how an individual may experience bullying and its consequences differently, depending on the context of that bullying event or situation. A “person by situation by context” interaction has been applied to research in other areas, and the recent National Academies of Science, Engineering, and Medicine report supports integration of these frameworks into research on bullying [21]. This study’s findings provide a conceptual model to understand an individual’s journey through these experiences, but further work is needed to understand how context plays a role in determining outcomes of a bullying event or experience.

### Limitations

Several limitations to this study should be considered. Traditional concept mapping methodology provides guidelines for small numbers of participants at each stage. To provide additional depth to this process, we included a larger number of participants than is typically involved in concept mapping to represent the various stakeholders who are involved in cyberbullying. As we used a purposeful sample, this study’s participants are not generalizable, and they may have had similar perspectives. However, this study’s findings that many participants provided insights and quotations from direct experience with cyberbullying support our purposeful sampling approach to engage a stakeholder involved in cyberbullying situations. Furthermore, the majority of data collection was focused in 1 geographic location. This study focused on cyberbullying applied to adolescents; we did not specifically target or include cyberbullying as applied to children or adults. Further work should investigate whether findings may generalize to young-adult age groups, in which cyberbullying has been shown to be common [22,23]. Finally, we modified the traditional concept mapping approach by adding a translation to the conceptual model step using key informant interviews. This step adds value to this study’s outcomes, but this is by definition outside the typical 5-step concept mapping process.

### Implications

Despite these limitations, this study has important implications in illustrating the key factors that define cyberbullying from the perspectives of stakeholders. The conceptual model developed in this project illustrates what key factors have been internalized by stakeholders both through direct experience and through exposure to sources, such as schools, media, and patients. The arrangement of concepts in our conceptual model suggests that cyberbullying cannot be considered a distinct entity from bullying, which is supported by the recent National Academies report [21]. However, stakeholders perceive that there are aspects of cyberbullying that support it as more than just another bullying context. Although the Uniform Definition of bullying was created to apply to bullying across all types and contexts, this study illustrates that there is still a strong public perception that cyberbullying presents distinct opportunities and challenges compared with traditional constructs of bullying. To unify efforts to prevent and intervene with bullying, as well as to measure and assess it over time, future work must address these stakeholder perceptions. To promote acceptance and uptake of the Uniform Definition of bullying among stakeholders, it is possible that the definition would benefit from evaluation for the context of cyberbullying or consideration toward adding language to clarify its application to cyberbullying. This study’s findings suggest that clarifications to the Uniform Definition may include acknowledgment that power imbalance may be created by tools such as the internet rather than cyberbullying being solely considered as a preexisting condition within a perpetrator. As the Uniform Definition is meant to be used by educators, policy makers, and researchers in the realms of both traditional bullying and cyberbullying, it is important to ensure consistency in interpretation and application of this definition across these stakeholder groups. Implications for policy include providing clarity and consistency in language when measuring bullying and in policies that address bullying. Assessment tools may need to clarify whether questions about bullying behavior include cyberbullying, and they may need to use consistent terms and language to ensure responses are valid across populations and over time. Furthermore, most states currently have separate policies for addressing bullying and cyberbullying, which may contribute to public perceptions that these represent separate entities [21]. This study’s findings support that integration of these concepts is supported by the overlap in key concepts. Implications for health providers working with teens and their parents include understanding that assessments for bullying need to address both traditional bullying and cyberbullying. Asking questions in a clinic that encompass both experiences or are open ended—such as “have you ever had any experiences with bullying?—may promote open discussion about the different types of bullying or different roles a teen may have played. Furthermore, providers can acknowledge that experiencing either or both types of bullying is common and consequential. Fortunately, newer studies suggest that interventions designed to address cyberbullying also affect bullying, further illustrating the strong connection across bullying behavior [20,24].

### Conclusions

In conclusion, findings support that cyberbullying is best understood in the broader context of bullying, but findings also support that stakeholder perceptions about the uniqueness of cyberbullying are strong. Bullying presents a complex set of behavior within roles that may be fluid and may lead to negative consequences for both perpetrators and targets. Findings may be applied toward achieving greater consistency in our definitions, assessments, and policies regarding bullying, and findings may be applied toward working toward a shared understanding of key concepts in bullying with stakeholders who are in the field, addressing bullying with teens and their parents as part of their everyday jobs.

## Abbreviations

CDC: Centers for Disease Control and Prevention
MDS: multidimensional scaling
SSSM: square symmetric-similarity matrix
